# Anti-Diabetic Activity of a Novel Exopolysaccharide Produced by the Mangrove Endophytic Fungus *Penicillium janthinellum* N29

**DOI:** 10.3390/md21050270

**Published:** 2023-04-26

**Authors:** Zhuling Shao, Yingying Tian, Shan Liu, Xiao Chu, Wenjun Mao

**Affiliations:** 1 Key Laboratory of Marine Drugs of Ministry of Education, Shandong Provincial Key Laboratory of Glycoscience and Glycotechnology, School of Medicine and Pharmacy, Ocean University of China, Qingdao 266003, China; 2 Marine Biomedical Research Institute of Qingdao, Qingdao 266237, China; 3 Laboratory for Marine Drugs and Bioproducts, National Laboratory for Marine Science and Technology (Qingdao), Qingdao 266237, China

**Keywords:** mangrove endophytic fungus, exopolysaccharide, structure, anti-diabetic activity

## Abstract

Marine microorganisms often produce exopolysaccharides with novel structures and diverse biological activities due to their specific marine environment. The novel active exopolysaccharides from marine microorganisms have become an important research area in new drug discovery, and show enormous development prospects. In the present study, a homogeneous exopolysaccharide from the fermented broth of the mangrove endophytic fungus *Penicillium janthinellum* N29, designated as PJ1-1, was obtained. The results of chemical and spectroscopic analyses showed that PJ1-1 was a novel galactomannan with a molecular weight of about 10.24 kDa. The backbone of PJ1-1 was composed of →2)-α-d-Man*p*-(1→, →4)-α-d-Man*p*-(1→, →3)-β-d-Gal*f*-(1→ and →2)-β-d-Gal*f*-(1→ units with partial glycosylation at C-3 of →2)-β-d-Gal*f*-(1→ unit. PJ1-1 had a strong hypoglycemic activity in vitro, evaluated using the assay of α-glucosidase inhibition. The anti-diabetic effect of PJ1-1 in vivo was further investigated using mice with type 2 diabetes mellitus induced by a high-fat diet and streptozotocin. The results indicated that PJ1-1 markedly reduced blood glucose level and improved glucose tolerance. Notably, PJ1-1 increased insulin sensitivity and ameliorated insulin resistance. Moreover, PJ1-1 significantly decreased the levels of serum total cholesterol, triglyceride and low-density lipoprotein cholesterol, enhanced the level of serum high-density lipoprotein cholesterol and alleviated dyslipidemia. These results revealed that PJ1-1 could be a potential source of anti-diabetic agent.

## 1. Introduction

Diabetes mellitus is a kind of endocrine metabolic disease, and its incidence has recently increased because of changes in diet, unhealthy lifestyles and environmental factors [1]. Type 2 diabetes mellitus (T2DM) is the most common type of diabetes around the world and accounts for about 90% of diabetes patients [2]. T2DM is characterized by hyperglycemia that will lead to a series of complications, including hyperlipidemia, diabetic nephropathy and liver impairment [3]. Diabetic complications can cause serious damage to human health and even threaten life [4,5]. Anti-diabetes drugs, such as metformin, rosiglitazone, sulfonylurea, acarbose and miglitol, have been widely used in the therapy of diabetes [6]. However, the drugs possess some side effects, such as hepatotoxicity and adverse gastrointestinal symptoms [7]. In order to meet the growing demand for anti-diabetic drugs, it is crucial to search for natural sources of anti-diabetic agents.

Polysaccharides have attracted considerable attention due to their unique structures and biological activities. It has been noted that they have potential anti-diabetic activity [8]. Sun et al. found that the exopolysaccharide EPS-III produced by *Cordyceps militaris* effectively inhibited α-glucosidase activity, and it could reduce plasma glucose concentration, improve glucose tolerance and repair dyslipidemia in STZ-induced diabetic mice [9]. The polysaccharides isolated from *Gomphidiaceae rutilus* increased insulin sensitivity and reduced blood glucose level [10]. Ye et al. revealed that the polysaccharides from *Enteromorpha prolifera* improved oral glucose tolerance metric and relieved insulin resistance in T2DM mice [11]. Shan et al. reported that the fucoidan from *Fucus vesiculosus* possessed a potent inhibitory effect on α-glucosidase activity [12]. The stimulatory activity of polysaccharides from algae on insulin secretion in vitro was also found [13]. So far, investigation into the anti-diabetic properties of the exopolysaccharides from mangrove endophytic fungi has rarely been reported.

Mangrove forests are considered to be dynamic ecotones or transition zones between terrestrial and marine habitats, and are biodiversity spots for marine fungi [14]. Mangrove fungi constitute the second largest ecological group of marine fungi due to their complex and special environment [15]. However, few reports have correlated the structure and biological activity of exopolysaccharides from mangrove endophytic fungi. In this study, a homogenous exopolysaccharide which possessed an obvious hypoglycemic activity in vitro was first isolated from the liquid culture broth of the mangrove endophytic fungus *Penicillium janthinellum* N29. The structure of the exopolysaccharide was characterized by a combination of chemical and spectroscopic methods, and its anti-diabetic effect in vivo was investigated.

## 2. Results and Discussion

### 2.1. Structural Characteristics of the Exopolysaccharide PJ1-1

**Figure 1:**
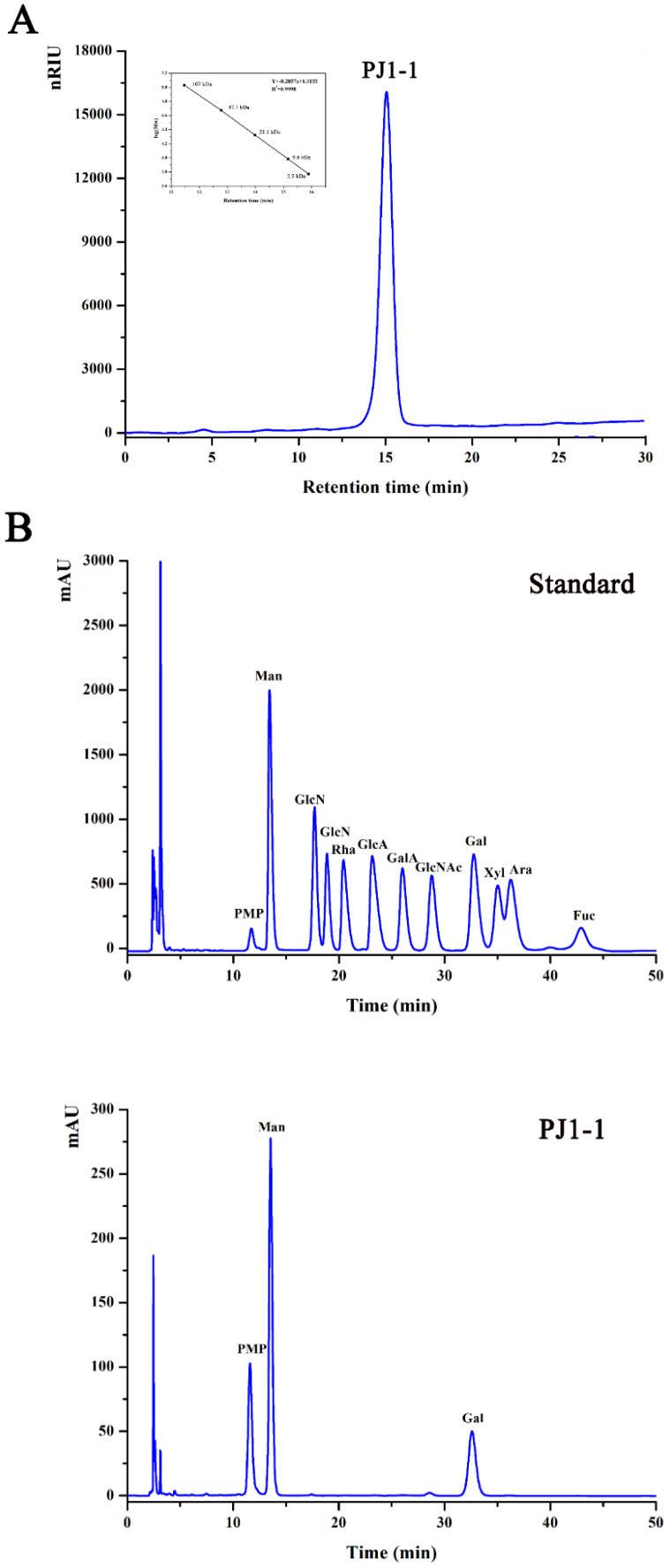

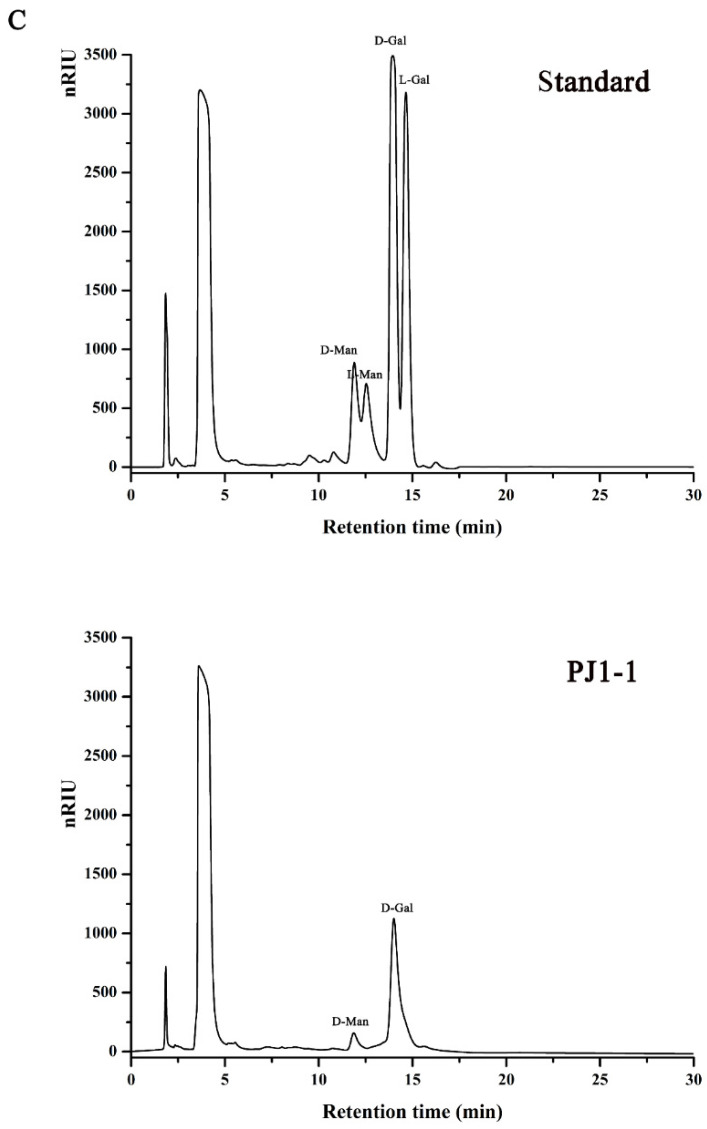
High-performance gel permeation chromatography and high-performance liquid chromatography chromatograms of PJ1-1. (**A**) High-performance gel permeation chromatography chromatogram on a Shodex OHpak SB-803 HQ column and the standard curve of molecular weight; (**B**) high-performance liquid chromatography chromatogram for the monosaccharide composition analysis (Man: d-mannose, GlcN: d-glucosamine, Rha: l-rhamnose, GlcA: d-glucuronic acid, GalA: d-galacturonic acid, Glc: d-glucose, Gal: d-galactose, Xyl: d-xylose, Ara: l-arabinose, Fuc: l-fucose); and (**C**) high-performance liquid chromatography chromatogram for the sugar configuration determination (d-Man: d-mannose, l-Man: l-mannose, d-Gal: d-galactose, l-Gal: l-galactose).

The crude exopolysaccharide was obtained from the culture medium of the mangrove endophytic fungus *P. janthinellum* N29. The yield of the crude exopolysaccharide was about 6.25 g/L. The exopolysaccharide was fractionated using a Q Sepharose Fast Flow column into three fractions. The fraction eluted with distilled water was the most abundant and was further purified on a Sephacryl S-100/HR column. A major fraction, designated PJ1-1, was obtained. PJ1-1 displayed a single and symmetrical peak in the high-performance gel permeation chromatography (HPGPC) chromatogram (Figure 1A); thus, PJ1-1 was a homogeneous polysaccharide. The molecular weight of PJ1-1 was estimated to be about 10.24 kDa by reference to a calibration curve made by pullulan standards. The total sugar content of PJ1-1 was 97.45%. No protein was detected in PJ1-1. The ultraviolet (UV) spectrum analysis of PJ1-1 showed that no absorption peak appeared at 280 nm, further illustrating that PJ1-1 did not contain protein [16]. Reversed-phase high-performance liquid chromatography (HPLC) analysis showed that PJ1-1 consisted of mannose (52.71%) and galactose (47.29%) (Figure 1B). The sugar configuration analysis using HPLC indicated that both the mannose and galactose in PJ1-1 were in the d-configuration (Figure 1C). 

Methylation analysis can provide important information regarding the linkage pattern of sugar residues. The identification and the proportion of the methylated alditol acetate of PJ1-1 are listed in Table 1. The mannose part of PJ1-1 showed three ion peaks. 1,5-Di-*O*-acetyl-2,3,4,6-tetra-*O*-methyl mannitol was detected, indicating the presence of the terminal unit Man*p*-(1→, 1,2,5-Tri-O-acetyl-3,4,6-tri-*O*-methyl mannitol and 1,4,5-tri-*O*-acetyl-2,3,6-tri-*O*-methyl mannitol were attributed to →2)-Man*p*-(1→ and →4)-Man*p*-(1→ residues, respectively. The galactose part of PJ1-1 had four ion peaks. 1,4-Di-*O*-acetyl-2,3,5-tri-*O*-methyl-galactitol was detected, indicating the presence of the terminal unit Gal*f*-(1→. 1,2,4-Tri-*O*-acetyl-3,5,6-di-*O*-methyl galactitol likely originating from →2)-Gal*f*-(1→ residue, while 1,3,4-tri-*O*-acetyl-2,5,6-di-*O*-methyl galactitol could represent →3)-Gal*f*-(1→ residue. 1,2,3,4-Tetra-*O*-acetyl-5,6-*O*-methyl galactitol could be from →2,3)-Gal*f*-(1→ residue. The presence of →2,3)-Gal*f*-(1→ suggested that PJ1-1 contained partial branches at C-2 of →3)-Gal*f*-(1→ or C-3 of→2)-Gal*f*-(1→ residues. The linkage patterns of the mannose and galactose units were further confirmed by NMR analysis. 

**Figure 2:**
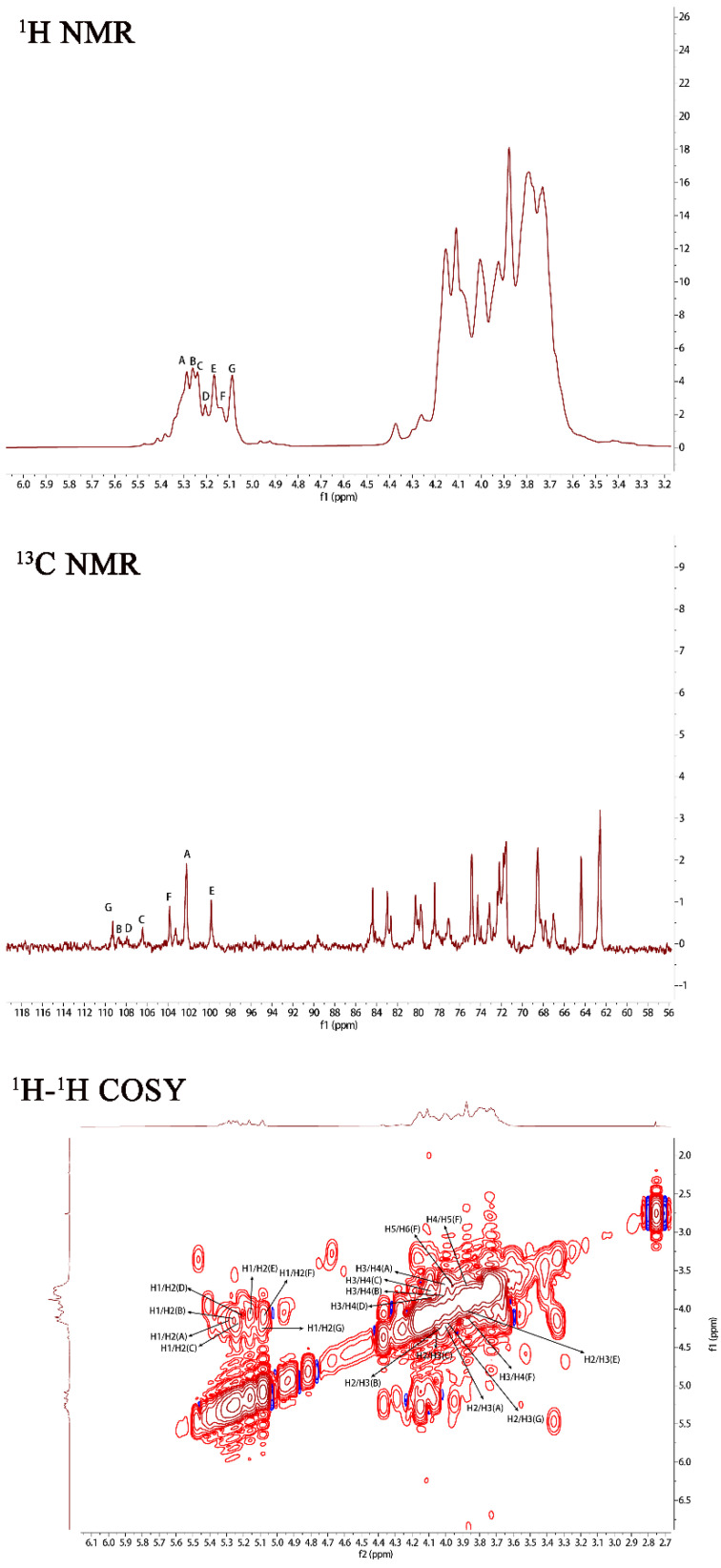

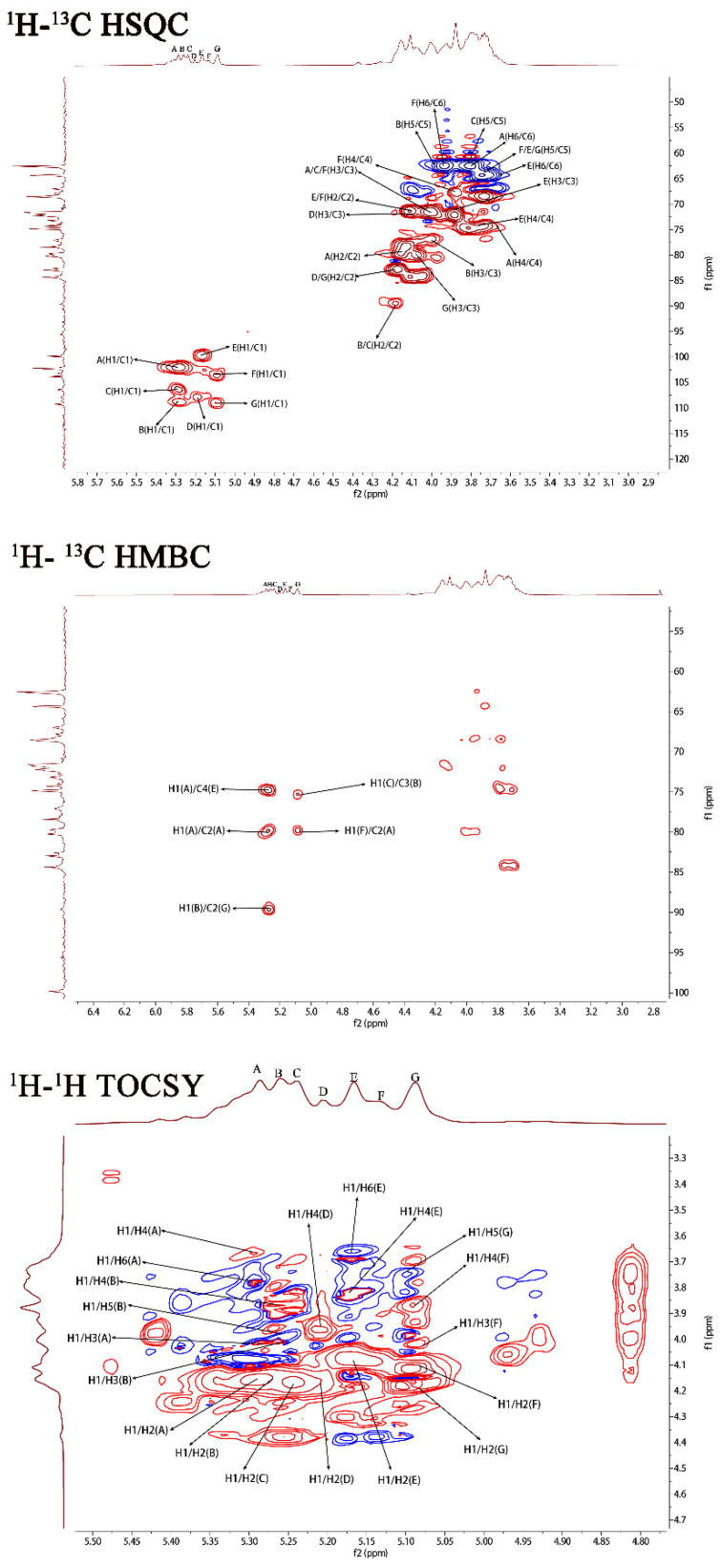

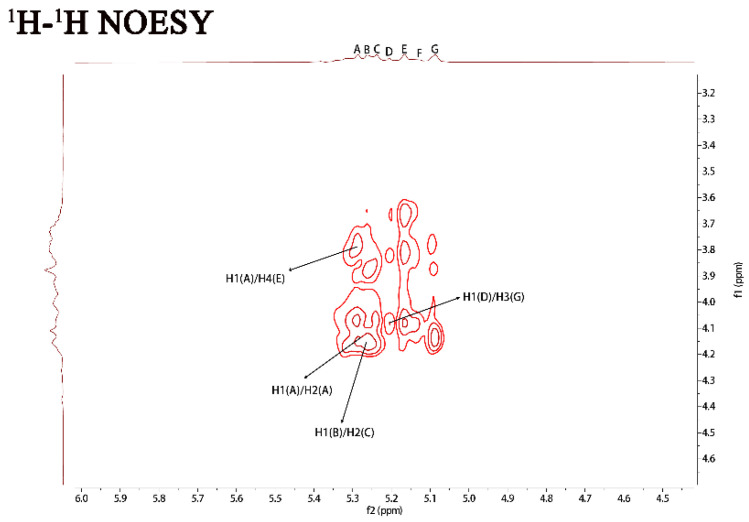
NMR spectra of PJ1-1. Spectra were performed on an Agilent DD2 500 MHz NMR spectrometer using acetone as internal standard. A: →2)-α-D-Man*p*-(1→; B: →2,3)-β-D-Gal*f*-(1→; C: →2)-β-D-Gal*f*-(1→; D: β-D-Gal*f*-(1→; E: →4)-α-D-Man*p*-(1→; F: α-D-Man*p*-(1→; and G: →3)-β-D-Gal*f*-(1→. Man*p*: mannopyranose, Gal*f*: galactofuranose.

In order to further investigate the structure of PJ1-1, NMR spectra analysis of PJ1-1 was carried out (Figure 2). In the ^1^H NMR spectrum of PJ1-1, 7 anomeric proton signals, which occurred at 5.29, 5.25, 5.23, 5.20, 5.16, 5.12 and 5.08 ppm, had relative integrals of 1.92:0.75:0.85:0.49:0.99:0.53:1. Other proton signals were located in the region of 4.20–3.60 ppm, which were attributed to H2–H6 of the sugar residues. In the anomeric region of the ^13^C NMR spectrum of PJ1-1, 7 anomeric carbon signals appeared at 99.76, 102.19, 103.79, 106.25, 107.87, 108.67 and 109.31 ppm. The chemical displacement of heterocarbon (C1) corresponds to α-type and β-type glucoside structures in the ^13^C NMR spectra at 90–104 ppm and 105–110 ppm, respectively [17]. The anomeric carbon signals at 106.25, 107.87, 108.67 and 109.31 ppm illustrated the presence of β-galactofuranose residues in PJ1-1 because only the anomeric carbon signals of β-d-galactofuranose and α-l-arabinofuranose could move to more than 105 ppm in the low field [18]. The signals at 99.76, 102.19 and 103.79 ppm were attributed to α-mannopyranose units [19,20]. The C2–C6 signals were distributed in the region of 60–90 ppm. The chemical shifts at 81.42 and 89.53 ppm might have been the C2–C4 signals of β-d-galactofuranose as the chemical shifts of C2–C6 would not exceed 80 ppm in common hexopyranose [21]. 

The ^1^H–^1^H correlated spectroscopy (COSY) and ^1^H–^1^H total correlation spectroscopy (TOCSY) afforded a variety of proton correlations with the sugar units. The C–H correlations were assigned from the ^1^H–^13^C heteronuclear single quantum coherence spectroscopy (HSQC) of PJ1-1. The H-1 of A at 5.29 ppm was related with the C-1 at 102.19 ppm, and A was assigned to →2)-α-d-Man*p*-(1→ unit due to the downfield shift of C-2 (79.67 ppm) in comparison with that of the parent α-d-Man*p* [22]. The H-1 of B at 5.25 ppm corresponded to the C-1 at 108.67 ppm. B was attributed to →2,3)-β-d-Gal*f*-(1→ unit because the signals of the down-field chemical shifts of C-2 at 89.53 ppm and C-3 at 75.87 ppm [23]. The H-1 of C at 5.23 ppm was related to the C-1 at 106.25 ppm. C was assigned to the →2)-β-d-Gal*f*-(1→ unit because the C-2 shift of C changed to low displacement at 89.53 ppm [24]. The H-1 signal of D at 5.20 ppm was correlated to the anomeric carbon signal at 107.87 ppm, and D was assigned to the β-d-Gal*f*-(1→ unit [25]. The H-1 signal of E at 5.16 ppm was related to the C-1 signal at 99.76, and E was attributed to the →4)-α-d-Man*p*-(1 → unit due to the correlated signals H-4/C-4 (3.82/74.78 ppm). The H-1 signal of F at 5.12 ppm was correlated to the C-1 signal at 103.79 ppm, and F was assigned to the α-d-Man*p*-(1→ unit [26]. G was assigned to the →3)-β-d- Gal*f*-(1 → unit due to the shift of the C-3 changing to low displacement at 79.78 ppm. By combining the data from the ^1^H–^1^H COSY, ^1^H–^1^H TOCSY and ^1^H–^13^C HSQC spectra, the assignment of the main proton and carbon signals of the seven sugar residues could be completed (Table 2). 

The repeating sequences in PJ1-1 were obtained using ^1^H–^1^H nuclear Overhauser enhancement spectroscopy (NOESY) and ^1^H–^13^C heteronuclear multiple bond correlation spectroscopy (HMBC). In the ^1^H–^13^C HMBC spectrum, the cross signal H-1(B)/C-2(C) proved that the C-1 of the →2,3)-β-d-Gal*f*-(1→ unit was linked to the O-2 position of the →2)-β-d-Gal*f*-(1→ unit. The cross signal H-1(A)/C-2(A) illustrated that the C-1 of the →2)-α-d-Man*p*-(1→ unit was attached to the O-2 position of the →2)-α-d-Man*p*-(1→ unit. The cross signal H-1(A)/C-4(E) showed that the C-1 of the →2)-α-D-Man*p*-(1→ unit was linked to the O-4 position of the →4)-α-D-Man*p*-(1→ unit. The related signal H-1(F)/C-2(A) indicated that the C-1 of the α-d-Man*p*-(1→ unit was attached to the O-2 position of the →2)-α-d-Man*p*-(1→ unit. The cross signal H-1(G)/C-3(B) proved that the C-1 of the →3)-β-d-Gal*f*-(1→ unit was linked to the O-3 position of the →2,3)-β-d-Gal*f*-(1→ unit. Furthermore, the cross signal H-1 (G)/C-3 (B) revealed the characteristics of the side chain. In the ^1^H–^1^H NOESY spectrum, the related signal H-1(D)/H-3(G) proved that the C-1 of the β-d-Gal*f*-(1→ unit was linked to the O-3 position of the →3)-β-d-Gal*f*-(1→ unit. The cross signals H-1(A)/H-4(E), H-1(A)/H-2(A) and H-1(B)/H-2(C) further proved the presence of sequences →2)-α-D-Man*p*-(1→4)-α-D-Man*p*-(1→, →2)-α-D-Man*p*-(1→2)-α-D-Man*p*-(1→ and →2,3)-β-d-Gal*f*-(1→2)-β-d-Gal*f*-(1→.

**Figure 3:**
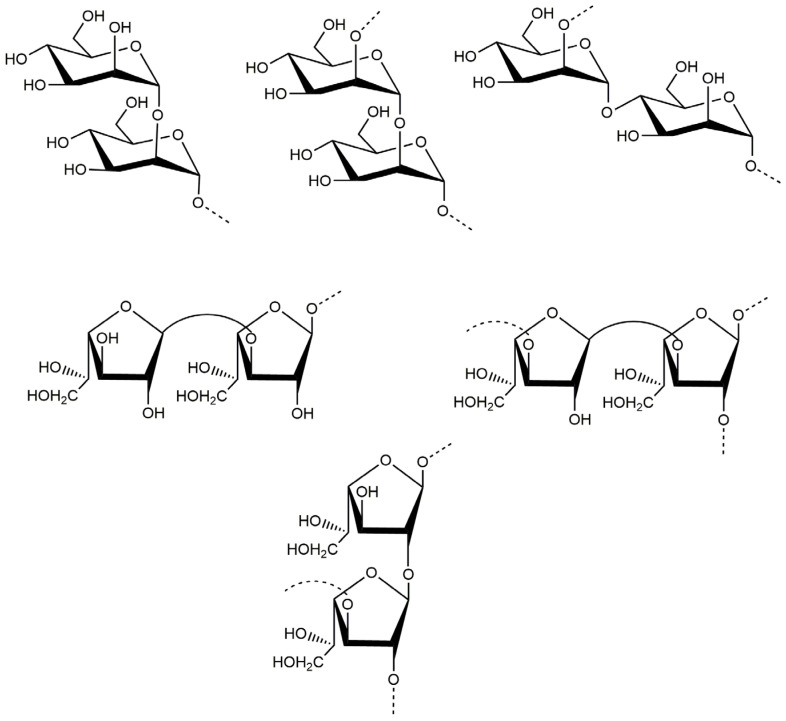
Possible structures of the main repeating disaccharides in PJ1-1.

Based on the above analyses, it was concluded that the backbone of PJ1-1 was constituted by the →2)-α-d-Man*p*-(1→, →4)-α-d-Man*p*-(1→, →3)-β-d-Gal*f*-(1→ and →2)-β-d-Gal*f*-(1→ units with partial branches at C-3 of the →2)-β-d-Gal*f*-(1→ unit. The branches were mainly composed of the →3)-β-d-Gal*f*-(1→ unit. An in-depth spectroscopic investigation on the fine structure of the side chains in PJ1-1 is required. The possible major disaccharides in PJ1-1 was shown in Figure 3.

So far, reports on the structural characterization of exopolysaccharides from the mangrove endophytic fungus have been seldom found. The exopolysaccharide Fw-1 from the mangrove-associated fungus *Fusarium oxysporum* contained a backbone of (1 → 6)-linked β-d-galactofuranose residue, and the branches were constituted by terminal α-d-glucopyranose residue, or short chains containing (1 → 2)-linked α-d-glucopyranose, (1 → 2)-linked β-d-mannopyranose and terminal β-d-mannopyranose residues. The side chains were connected to the C-2 of the galactofuranose residue of the backbone [27]. The backbone of the exopolysaccharide As1-1 from the mangrove endophytic fungus *Aspergillus* sp. Y16 mainly consisted of a (1→2)-linked α-d-mannopyranose unit, substituted at C-6 by the (1→6)-linked α- d -mannopyranose, (1→)-linked β-d-galactofuranose and (1→)-linked β-d-mannopyranose units [28]. PJ1-1 possessed different structural characteristics from the exopolysaccharides from these mangrove endophytic fungi. The exopolysaccharide PJ1-1 from the mangrove endophytic fungus *P. janthinellum* N29 constituted the →2)-α-d-Man*p*-(1→, →4)-α-d-Man*p*-(1→, →2)-β-d-Gal*f*-(1→ and →3)-β-d-Gal*f*-(1→ units. The branch which contained the →3)-β-d-Gal*f*-(1→unit was at C-3 of the →2)-β-d-Gal*f*-(1→ unit. The exopolysaccharides with galactofuranose units are often produced by mangrove endophytic fungi, and can be very useful chemotaxonomic markers [29]. Our result also demonstrated that the galactofuranose units may be a characteristic component of the exopolysaccharides from mangrove endophytic fungi. The generality extent of the structural characteristics in the exopolysaccharides from other mangrove endophytic fungi must be further investigated.

### 2.2. Influence of PJ1-1 on α-Glucosidase Activity In Vitro

**Figure 4:**
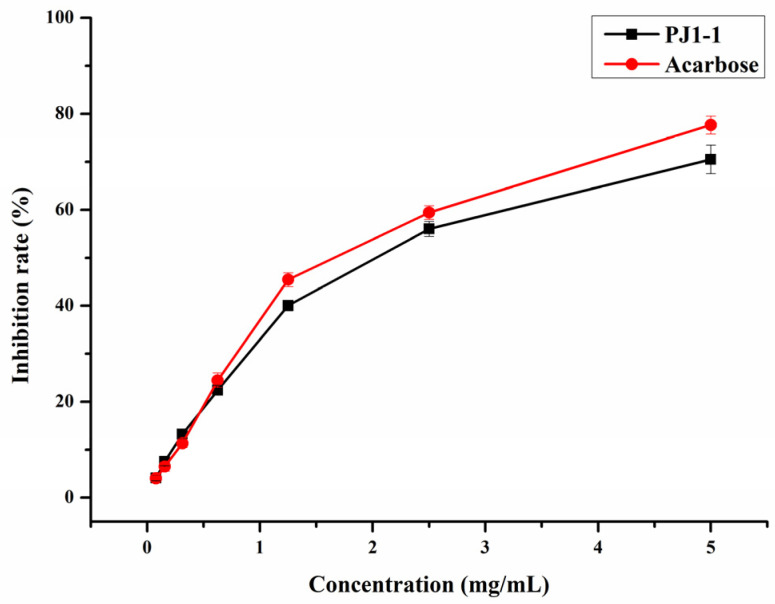
Inhibitory effect of PJ1-1 on α-glucosidase activity in vitro.

α-Glucosidase is a glycoside hydrolase found in the small intestine that may rapidly hydrolyze dietary starches and cause a rise in blood glucose level. Thus, the inhibition of α-glucosidase activity is an efficient strategy to reduce hyperglycemia in diabetic individuals. In the present study, the inhibition effect of PJ1-1 on α-glucosidase activity was investigated using acarbose as a reference. As shown in Figure 4, the inhibitory effect of PJ1-1 on α-glucosidase activity was in a concentration-dependent manner. The α-glucosidase activity was effectively inhibited by PJ1-1, and the inhibitory rate was about 70.52% at 5 mg/mL. Additionally, the inhibitory effect of PJ1-1 on α-glucosidase activity was slightly lower than that of acarbose. The data indicated that PJ1-1 could be a potential hypoglycemic polysaccharide. Thus, the anti-diabetic activity of PJ1-1 in vivo was further explored using T2DM mice induced by high-fat diet and STZ.

### 2.3. Antidiabetic Activity In Vivo of PJ1-1 

A combination of high-fat diet and low-dose streptozotocin (STZ) treatment has been extensively utilized to produce an experimental mice model with T2DM [30]. Feeding a high-fat diet to mice can result in blood glucose and serum lipid metabolic disorders, as well as insulin resistance, which are similar to the early symptoms of human T2DM. STZ injection is used to kill a portion of the islet β-cells. In this study, mice with T2DM induced by a high-fat diet and STZ were used to study the anti-diabetic effect of PJ1-1 in vivo.

#### 2.3.1. Effects of PJ1-1 on Body Weight and Fasting Blood Glucose Level

**Figure 5:**
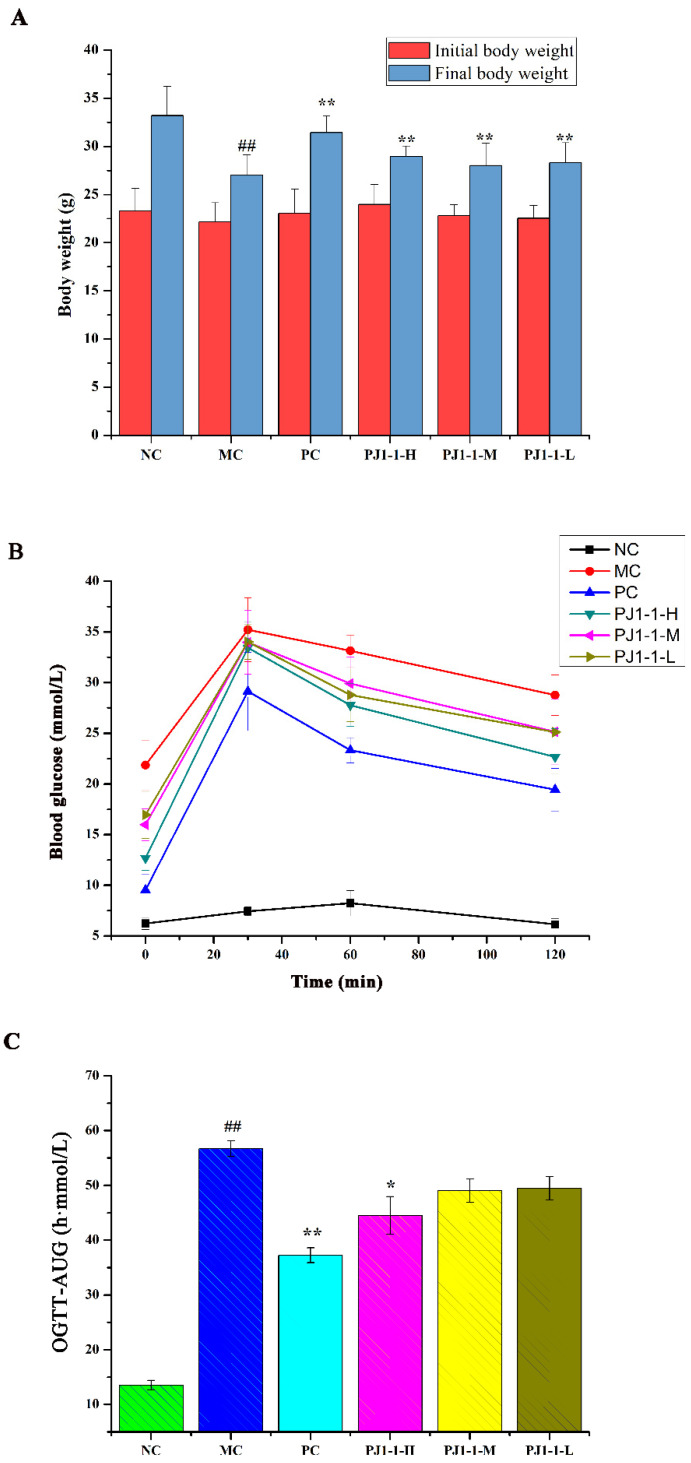
Effects of PJ1-1 on body weight and glucose tolerance in the type 2 diabetes mellitus mice. (**A**) Body weight; (**B**) oral glucose tolerance tests; and (**C**) oral glucose tolerance tests -area under the curve. ^##^ *p* < 0.01 vs. the normal control group; * *p* < 0.05 vs. the model control group, ** *p* < 0.01 vs. the model control group.

The influence of PJ1-1 on body weight is shown in Figure 5A. By comparison with the normal control (NC) group, the body weight in the model control (MC) group obviously reduced. However, after treatment with PJ1-1, the body weights significantly elevated compared with the MC group (*p* < 0.01). These results indicated that PJ1-1 could effectively improve the loss of body weight in high-fat-diet- and STZ-induced T2DM mice.

Hyperglycemia is a major symptom of diabetes; thus, reducing fasting blood glucose (FBG) is the main target of treating diabetes. FBG is a fundamental indicator of blood glucose balance. The effect of PJ1-1 on the level of FBG was investigated using the T2DM mice, and the results are listed in Table 3. The level of FBG in the NC group was stable at 5.10–5.27 mmol/L throughout the administration period. However, the level of FBG in the MC group substantially increased compared with the NC group (*p* < 0.01), illustrating that the blood glucose balance was disordered because of the high-fat diet and STZ treatment. After treatment with PJ1-1 for one week, the levels of FBG in the PJ1-1 groups continued to increase. However, from the third to fifth weeks, the levels of FBG in the PJ1-1 groups significantly decreased in comparison with the MC group (*p* < 0.01). In the fifth week, compared with the MC group, the levels of FBG in the high dose of PJ1-1 (PJ1-1-H) group, middle dose of PJ1-1 (PJ1-1-M) group and low dose of PJ1-1 (PJ1-1-L) group decreased by 42.16%, 29.10% and 22.26%, respectively. The decreasing effect of PJ1-1 on the level of FBG was dose-dependent. The level of FBG in the positive control (PC) group showed a 55.17% decrease in the fifth week. These data indicated that PJ1-1 could effectively improve blood glucose balance.

#### 2.3.2. Effect of PJ1-1 on Glucose Tolerance

The influence of PJ1-1 on the glucose tolerance in the T2DM mice was evaluated using the assays of oral glucose tolerance tests (OGTT) and OGTT-area under the curve (OGTT-AUC). As shown in Figure 5B, after 30 min of administration, the blood glucose levels increased rapidly and reached the highest value in all groups, and then gradually decreased. The blood glucose level in the NC group returned to normal at 120 min, but the glucose level in the MC group remained at a high level of about 28.77 mmol/L. These results indicated that glucose tolerance was impaired seriously in the T2DM mice. However, by comparison with the MC group, the blood glucose levels in the three PJ1-1 groups markedly reduced at 120 min, especially in the PJ1-1-H group. The glucose level in the PJ1-1-H group decreased to 22.67 mmol/L, illustrating that a certain concentration of PJ1-1 could improve glucose tolerance. The blood glucose level in the PC group reduced to 19.44 mmol/L at 120 min. As shown in Figure 5C, compared with the NC group, the OGTT-AUC increased by 76.14% (*p* < 0.01) in the MC group, indicating that the glucose tolerance of the T2DM mice was retrograded severely. However, the level of OGTT-AUC in the PJ1-1-H group significantly reduced compared with the MC group (*p* < 0.05). In addition, it was obvious that rosiglitazone treatment induced a significant decrease in the level of OGTT-AUC in comparison with the MC group. These results indicated that PJ1-1 possessed a better ability to stimulate glucose metabolism, and improved glucose tolerance in the T2DM mice.

#### 2.3.3. Effect of PJ1-1 on Insulin Resistance

Insulin is a potent anabolic agent, promoting the cellular uptake, storage and synthesis of nutrients, while blocking nutrient breakdown and release into the circulation. Insulin secretion deficiency and insulin resistance are typical features of T2DM, which result in hyperglycemia and impaired glucose metabolism [31]. Here, the assays of the fasting insulin content, the quantitative insulin sensitivity check index (QUICKI), the index of homeostasis model assessment of insulin resistance (HOMA-IR) and the index of homeostasis model assessment β (HOMA-β) were used for the evaluation of insulin resistance. 

**Figure 6:**
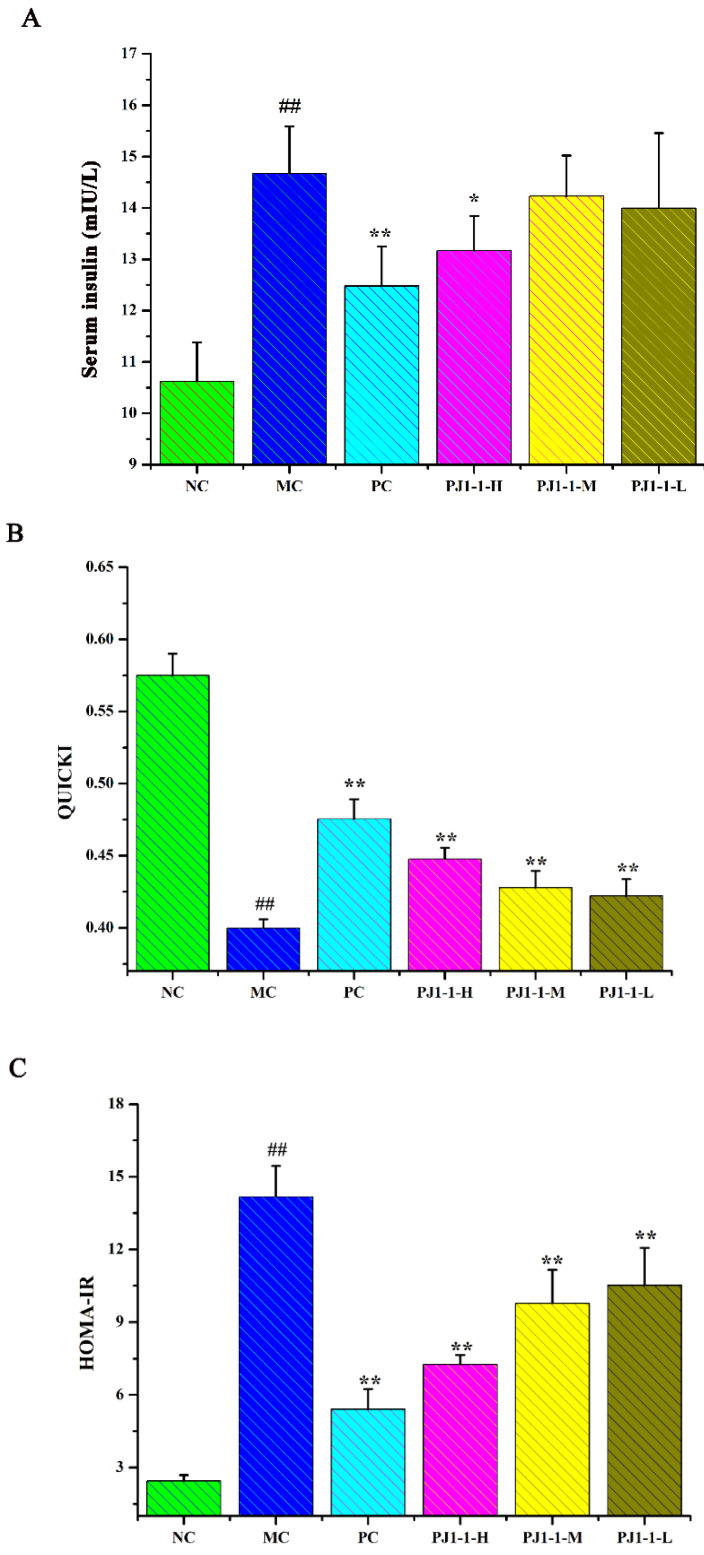

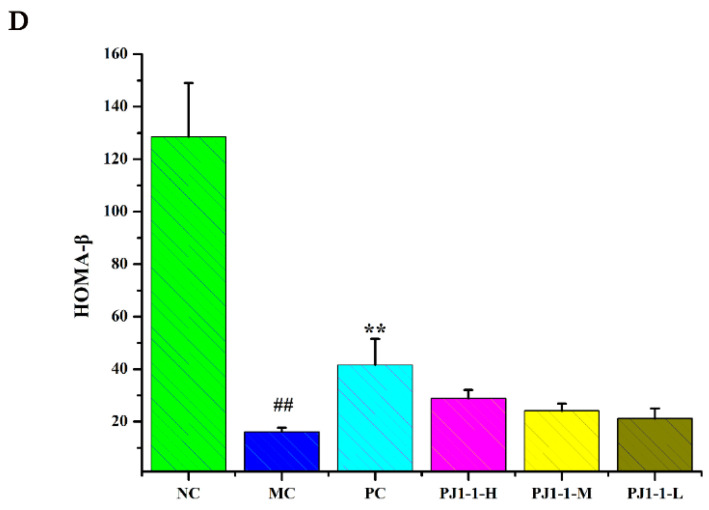
Influence of PJ1-1 on insulin resistance in the type 2 diabetes mellitus mice. (**A**) Fasting insulin content; (**B**) quantitative insulin sensitivity check index; (**C**) index of homeostasis model assessment of insulin resistance; and (**D**) index of homeostasis model assessment β. ^##^ *p* < 0.01 vs. the normal control group; * *p* < 0.05 vs. the model control group, ** *p* < 0.01 vs. the model control group.

As shown in Figure 6, after treatment with a high-fat diet and STZ, compared with the NC group, the fasting insulin content and HOMA-IR index in the MC group obviously increased, while the QUICKI and HOMA-β indexes obviously decreased, indicating that the diabetic mice occurred insulin resistance. However, compared with the MC group, the fasting insulin content in the PJ1-1-H group significantly decreased (*p* < 0.05). The fasting insulin content in the PJ1-1 m and PJ1-1-L groups also reduced, although these data were not significantly different compared with that of the MC group. The fasting insulin content in the PC group markedly diminished compared with the MC group (*p* < 0.01). It was also noted that PJ1-1 could markedly increase the QUICKI index compared with the MC group. The indexes of QUICKI in the PJ1-1-H, PJ1-1 m and PJ1-1-L groups increased by 12.80%, 7.69% and 7.66%, respectively. For the PC group, the index of QUICKI increased by 20.51%. Additionally, PJ1-1 significantly decreased the HOMA-IR index in a dose-dependent manner compared with the MC group, but PJ1-1 did not exhibit a noticeable effect on the HOMA-β index in comparison with the MC group. The QUICKI index is a critical indicator that measures insulin sensitivity in mice. The higher the content, the greater the carbohydrate breakdown efficiency. HOMA-IR reflects the hypoglycemic efficiency of insulin in mice due to the feedback loop between the blood glucose output and insulin secretion. The responsiveness of the liver and peripheral tissues to insulin reduced as the HOMA-IR score rose. These results indicated that PJ1-1 could effectively increase insulin sensibility, ameliorate insulin resistance and improve the hypoglycemic efficiency of insulin in the T2DM mice.

#### 2.3.4. Influences of PJ1-1 on Lipid Metabolism

**Figure 7:**
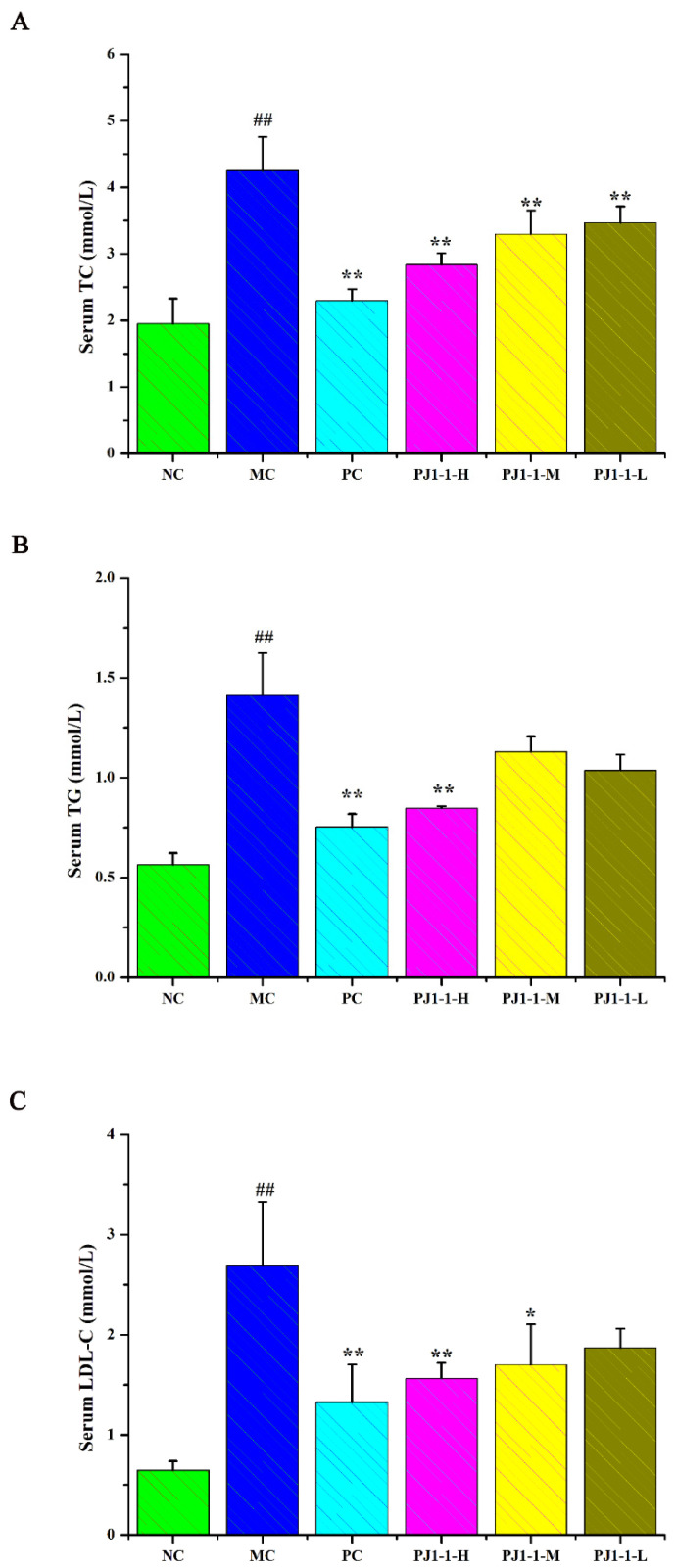

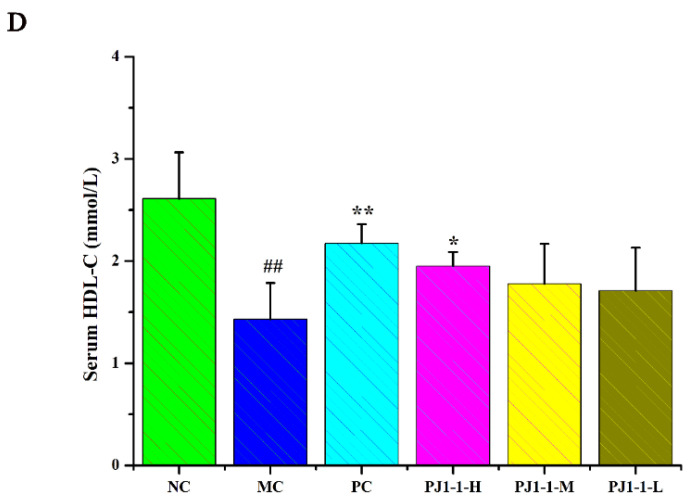
Effect of PJ1-1 on lipid metabolism in the type 2 diabetes mellitus mice. (**A**) Total cholesterol level; (**B**) triglyceride level; (**C**) low-density lipoprotein-cholesterin level; and (**D**) high-density lipoprotein–cholesterin level. ^##^ *p* < 0.01 vs. the normal control group; * *p* < 0.05 vs. the model control group, ** *p* < 0.01 vs. the model control group.

Dyslipidemia is one of the risk factors of T2DM and causes a disruption in lipid metabolism. Hyperlipidemia is an early event in the development of cardiovascular disease in T2DM patients. Dysregulation of lipid metabolism can result in systemic disruption of insulin and glucose metabolism [32]. As shown in Figure 7, compared with the NC group, the total cholesterol (TC), triglyceride (TG) and low-density lipoprotein-cholesterin (LDL-C) levels in the MC group significantly increased (*p* < 0.01) and the high-density lipoprotein–cholesterin (HDL-C) level markedly reduced (*p* < 0.01). After treatment with PJ1-1 for 5 weeks, the TG level significantly decreased in the PJ1-1-H group (*p* < 0.01) compared with the MC group. Moreover, PJ1-1 markedly increased the TC levels in a dose-dependent manner compared with the MC group. The LDL-C levels effectively diminished in the PJ1-1-H (*p* < 0.01) and PJ1-1 m (*p* < 0.05) groups, while the HDL-C level significantly increased in the PJ1-1-H group (*p* < 0.05). In addition, it was obvious that rosiglitazone treatment induced a significant decrease in the levels of TG, TC and LDL-C, and resulted in an increase in the HDL-C level in comparison with the MC group. These results suggested that PJ1-1 ameliorated dyslipidemia in the T2DM mice and promoted lipid metabolism by decreasing TG, TC and LDL-C levels and increasing HDL-C level.

The above results demonstrated that PJ1-1 possesses an obvious anti-diabetic activity in vivo. PJ1-1 significantly improved blood glucose balance and glucose tolerance. In addition, PJ1-1 increased insulin sensitivity and ameliorated insulin resistance. Furthermore, PJ1-1 obviously reduced the serum TG, TC and LDL-C levels, increased HDL-C level and repaired dyslipidemia. Hyperglycemia is a major symptom of diabetes that can cause a series of complications. T2DM is characterized by the increasing death of β-cells and resultant insulin secretion impairment. Insulin resistance pertains to low-efficiency glucose utilization that renders insulin insensitivity in an organism. The obvious features of insulin resistance include high levels of blood glucose and serum insulin. Moreover, T2DM is frequently accompanied by aberrant lipid metabolism [33]. The present data revealed that PJ1-1 had the potential to develop into a novel anti-diabetic agent for prevention and therapy of T2DM. The anti-diabetic activity of PJ1-1 could be associated with its structural characteristics. The presence of α-(1→4) glycosidic linkages of polysaccharides has been reported to be critical for α-glucosidase inhibitory activity [34]. Gao et al. found that the polysaccharide ARLP-W from *Anoectochilus roxburghi*, which contained glycosidic linkages of β-1, 4-Man*p*, could decrease the level of hyperglycaemia, protect the islets, improve insulin resistance and increase the β-cell area in T2DM mice [35]. Liu et al. reported that the polysaccharides which possessed a β-d-(1→6)- glycosidic bond improved the insulin level and decreased the blood glucose level in streptozotocin-induced diabetic mice [36]. However, it is difficult to reach a conclusion on the relationship between the structure and antidiabetic activity of the polysaccharides. The generality extent of the relationship requires further investigation.

## 3. Materials and Methods

### 3.1. Materials

p-Nitrophenyl-α-d-glucopyranoside, α-glucosidase (EC 3.2.1.20, from Saccharomyces cerevisiae), 1-phenyl-3-methyl-5-pyrazolone and monosaccharide standards (d-mannose, l-rhamnose, d-glucose, d-glucuronic acid, d-galacturonic acid, N-acetyl-β-d-glucosamine, d-glucose, d-galactose, d-xylose, l-arabinose, and l-fucose) were obtained from Sigma–Aldrich (St. Louis, MO, USA). Pullulan standards (*Mw*: 5.9, 9.6, 21.1, 47.1, and 107 kDa) were obtained from Showa Denko K.K. (Tokyo, Japan). Q Sepharose Fast Flow and Sephacryl S-100/HR were obtained from GE Healthcare Life Sciences (Piscataway, NJ, USA). Bicinchoninic acid (BCA) protein assay kit and glucose assay kit with o-toluidine were obtained from Beyotime Biotechnology (Shanghai, China). STZ and rosiglitazone were obtained from Aladdin Chemical Co., Ltd. (Shanghai, China). Mouse insulin enzyme-linked immunosorbent assay (ELISA) kit was obtained from Solarbio Biotechnology (Beijing, China). The assay kits for TC, TG, HDL-C and LDL-C were obtained from Nanjing Jiancheng Bioengineering Institute (Nanjing, China). 

### 3.2. Animals

Four-week-old male C57BL/6J mice (18–22 g, SPF grade) were obtained from Charles River Laboratories (Beijing, China) and housed under standard feeding circumstances with a 12 h light/dark cycle and free access to food and water. Animal experiments were permitted by the institutional animal care and use committee of Ocean University of China (OUC-SMP-20220403). 

### 3.3. Strains and Culture Conditions

The fungus *P. janthinellum* N29 was isolated from a piece of fresh tissue from the inner part of the medicinal mangrove *Acanthus ilicifolius* collected from the South China Sea. It was identified according to a molecular biological protocol by DNA amplification and sequencing of the ITS region. The sequence data have been submitted to GenBank with the accession number MW178203. *P. janthinellum* N29 was grown in potato dextrose agar medium containing glucose (20 g/L), sea salt (15 g/L) and potato starch (200 g/L), at pH 7.0 at 28 °C for 15 days on a reciprocal shaker. Finally, 150 L of liquid culture broth was obtained.

### 3.4. Preparation of the Exopolysaccharide PJ1-1

The exopolysaccharide from the fermentation broth of the fungus *P. janthinellum* N29 was isolated according to the method reported previously [37]. The fermentation broth was centrifuged (8000× *g*, 10 min) to separate the mycelia and supernatant. The supernatant was concentrated to one-third of its original volume with a rotary evaporator under reduced pressure at 55 °C. Subsequently, 4 volumes of 95% ethanol were added (*v*/*v*) and kept at 4 °C for 24 h. The precipitate was collected via centrifugation (3600× *g*, 10 min) and dialyzed in a cellulose membrane tubing (flat width 44 mm, molecular weight cut off 3500) against distilled water for 48 h. The retained fraction was concentrated and lyophilized, and a crude polysaccharide was obtained. The crude polysaccharide was fractionated on a Q Sepharose Fast Flow column (60 cm × 30 mm) by eluting with a step-wise gradient of 0, 0.025 and 0.4 mol/L NaCl at a flow rate of 1.0 mL/min. The eluates were collected by an auto-collector, and the total sugar content was detected using the phenol–sulfuric acid method [38]. According to the profile of the gradient elution, the fraction PJ1 eluted with distilled water was the most abundant and was further purified on a Sephacryl S-100 column (95 cm × 25 mm) eluting with 0.2 mol/L NH_4_HCO_3_ at a flow rate of 0.2 mL/min. Major fractions were collected, freeze-dried and designated as PJ1-1.

### 3.5. Composition Analysis

Total sugar content was measured using the phenol–sulfuric acid method with mannose as the standard [38]. Protein was analyzed using the BCA protein assay kit [39]. Purity and molecular weight of PJ1-1 were assessed using HPGPC [40]. The assay was performed on an Agilent 1260 Infinity HPLC instrument (Agilent Technologies, Santa Clara, CA, USA) fitted with a Shodex OHpak SB-803 HQ column (8.0 mm × 300 mm, Showa Denko K.K., Tokyo, Japan) and a refractive index detector (Agilent RID-10A Series). The molecular weight was estimated by reference to a calibration curve made by pullulan standards (*Mw*: 107, 47.1, 21.1, 9.6 and 5.9 kDa). 

Monosaccharide composition was determined with reversed-phase HPLC after pre-column derivatization [41]. Briefly, PJ1-1 (5 mg) was hydrolyzed with 2 mol/L trifluoroacetic acid at 105 °C for 4 h. After that, the excess acid was removed with methanol using a nitrogen blower three times. The dry hydrolysate was dissolved in 1.5 mL of distilled water and centrifuged (4500× *g*, 10 min). Subsequently, the supernatant solution (160 μL) was derivatized with 160 μL of 0.3 mol/L NaOH, 400 μL of 0.5 mol/L PMP solution (in methanol) and 210 μL of distilled water at 70 °C for 60 min. After cooling down to room temperature, 160 μL of 0.3 mol/L HCl solution was added to terminate the reaction, followed by extraction with 0.8 mL of chloroform three times. The supernatant was filtered through a 0.45 μm membrane, and 10 μL of the resulting solution was injected into the Eclipse XDB-C18 column (4.6 mm × 250 mm, 5 μm). The chromatogram was performed on an Agilent 1260 Infinity HPLC instrument fitted with an Agilent XDB-UV detector (254 nm). The mobile phase was a mixture of 0.1 mol/L KH_2_PO_4_ in water (pH 6.7)–acetonitrile (83:17). The flow rate was 1.0 mL/min, and column temperature was 30 °C. 

Sugar configuration was measured as described by Tanaka et al. [42]. PJ1-1 was degraded with 2 mol/L trifluoroacetic acid at 105 °C for 4 h. Then, the hydrolysate was heated with l-cysteine methyl ester in pyridine at 60 °C for 1 h. The o-tolyl isothiocyanate solution was added to the mixture, and was further heated at 60 °C for 1 h. The reaction mixture was analyzed on an Agilent 1260 Infinity HPLC instrument fitted with an Eclipse XDB-C18 column and an Agilent XDB-UV detector (254 nm, Agilent Technologies, Santa Clara, CA, USA). 

### 3.6. Methylation Analysis

Methylation analysis of PJ1-1 was carried out according to the previously described method with some modification [43]. PJ1-1 (10 mg) was dried in a vacuum-drying oven (55 °C) for 24 h and then dissolved in 2 mL of anhydrous dimethyl sulfoxide with stirring for 12 h under nitrogen protection. Afterwards, 1 mL of iodomethane was added to the reaction system in the dark, N_2_ was used to drive out the air, and the reaction continued for another 1 h. The completion of methylation was confirmed by Fourier-transform infrared (FTIR) spectroscopy. The completely methylated product was re-dissolved in 2 mL of trifluoroacetic acid (2 mol/L) and sealed at 105 °C for 6 h, and then reduced with NaBH_4_ and acetylated to convert into alditol acetates by reacting with acetic anhydride–pyridine at 100 °C for 2 h. The acetylated product was dissolved in chloroform (4 mL) and washed with distilled water three times. Thereafter, the partially methylated alditol acetates were analyzed on a TRACE 1300-ISQ instrument (Thermo Scientific, Waltham, MA, USA) equipped with a DB-225 fused silica capillary column (0.25 mm × 30 m, 0.25 μm, Agilent Technologies, Santa Clara, CA, USA). 

### 3.7. Spectroscopy Analysis

^1^H NMR and ^13^C NMR spectra were performed at 25 °C on an Agilent DD2 500M NMR spectrometer (Agilent Technologies, Santa Clara, CA, USA). Polysaccharide (60 mg) was deuterium-exchanged by lyophilization three times with 99.9% D_2_O and then was dissolved in 0.5 mL of 99.9% D_2_O and transferred into NMR tube. Chemical shift was expressed as δ (ppm) relative to acetone (^1^H: 2.225 ppm, ^13^C: 31.07 ppm). ^1^H–^1^H COSY, ^1^H–^1^H TOCSY, ^1^H–^13^C HSQC, ^1^H–^1^H NOESY and ^1^H–^13^C HMBC experiments were also carried out. Spectra were processed and analyzed using MestReNova (V12.0.3, Mestrelab Research, Santiago de Compostela, Spain). For the FTIR spectrum, the polysaccharide was ground with KBr, pressed into a 1 mm transparent sheet and then determined on a Nicolet Nexus 470 spectrometer (Thermo Fisher Scientific, Waltham, MA, USA) with a scan range of 400–4000 cm^−1^. UV spectrum was recorded on a UV-2802 PC spectrophotometer (UNICO Shanghai Instrument Co., Ltd., Shanghai, China) between 190 and 400 nm.

### 3.8. α-Glucosidase Inhibitory Assay

The α-glucosidase inhibitory activity was measured as per the reported method with some modifications [44]. Summarily, the α-glucosidase (750 U) was dissolved in 0.01 mol/L PBS (2 mL, pH 6.8) and shaken for 5 min. The obtained α-glucosidase solution was applied to enzyme inhibition assays with an enzyme activity of 0.2 U/mL. A total of 90 μL of PJ1-1 with different concentrations (0.08, 0.16, 0.32, 0.64, 1.25, 2.50 and 5.00 mg/mL), 100 μL of α-glucosidase (0.5 U/mL) and 20 μL of p-nitrophenol-α-d-glucopyranoside substrate were prepared with 0.01 mol/L PBS (pH 6.8). The mixture solution was shaken vigorously and followed by incubation at 37 °C for 30 min. A total of 100 μL of 0.2 mol/L Na_2_CO_3_ was added to terminate the whole reaction. Acarbose was used as positive control. The absorbance was recorded at 405 nm using a microplate reader (Biotek ELx808, BioTek Instruments, Inc. Winooski, VT, USA). The α-glucosidase inhibitory rate was calculated as follows:

$$\mathrm{Inhibition}\;\mathrm{rate}\;(\%)=100\;-\;\frac{A_{\;test}-A_{\;test\;blank}}{A_{\;control}-A_{\;control\;blank}}\times 100$$

A*_test_*: PJ1-1 or acarbose. A*_test blank_*: PJ1-1 or acarbose, no α-glucosidase. A*_control_*: no PJ1-1 or acarbose. A*_control blank_*: no α-glucosidase and PJ1-1 or acarbose. The half inhibitory concentration value (IC_50_) was the concentration of the α-glucosidase inhibitor concentration which inhibited the α-glucosidase activity by 50%. 

### 3.9. In Vivo Experiment

#### 3.9.1. Animal Experimental Design

Four-week-old male C57BL/6J mice (18 ± 2 g) were housed in a room with a 12 h light/dark cycle, a temperature of 24–26 °C and a relative humidity of 50–70% with free access to food and water. After a one-week environmental adaptation period, six mice were randomly separated as the NC group and fed a normal diet. The other mice were fed a high-fat diet (10% lard oil, 20% saccharose, 2.5% cholesterol, 0.5% sodium cholate and 67% normal diet) for 6 weeks with sufficient food and water, and then were subjected to intraperitoneal injection of STZ at a dose of 30 mg/kg on day 1–3. After 3 days of STZ treatment, the FBG levels of the mice were measured using a glucose assay kit. The mice with FBG ≥ 11.1 mmol/L were regarded as diabetic and then used in the subsequent analysis.

The diabetic mice were randomly divided into five groups (*n* = 6 per group): MC group, PC group, PJ1-1-L group (100 mg/kg/day), PJ1-1 m group (200 mg/kg/day) and PJ1-1-H group (400 mg/kg/day). For the next 35 days, the mice in the PJ1-1 groups were treated with PJ1-1 (100, 200, or 400 mg/kg) via intragastric administration once a day, and the mice in the NC and MC groups were administrated with corresponding volumes of saline. Meanwhile, the mice in the PC group were treated with rosiglitazone at a dose of 200 mg/kg via intragastric administration once a day. The body weight and FBG level of the mice were measured once a week. Their water and food intake was monitored once every 3 days. After 12 h of the final administration, the mice were sacrificed via cervical dislocation. The body weights of mice were measured, and the blood samples were taken from the orbital sinus and centrifuged (4000× *g*, 10 min) to gain the serum samples, which were stored at –80 °C until analysis.

#### 3.9.2. FBG and OGTT 

All mice were fasted for 12 h every weekend and their FBG levels were measured using a glucose assay kit with o-toluidine [45]. After 35 days of intragastric administration, the 12 h fasted mice in all groups were intragastrically administered glucose (2.0 g/kg). The blood glucose levels were measured sequentially at 0, 30, 60 or 120 min. AUC was calculated according to the following formula:

AUC = 0.25 × (G _0h_ + G _0.5h_) + 0.25 × (G _0.5h_ + G _1.0h_) + 0.5 × (G _0h_ + G _2.0h_).

#### 3.9.3. Assays of Fasting Insulin Content and Related Indexes

Fasting insulin content of serum was measured using an ELISA kit according to the manufacturer’s instructions [46]. Additionally, the HOMA-IR index was calculated using the formula: HOMA-IR = FBG content (mmol/L) × fasting insulin content (mIU/L)/22.5. The HOMA-β index was calculated using the formula: HOMA-β = 20 × fasting insulin content (mIU/L)/[FBG (mmol/L) − 3.5]. QUICKI was measured using the following equation: 1/(lg FBG content (mmol/L) + lg fasting insulin content (mIU/L)) [47]. 

#### 3.9.4. Determination for Lipid Metabolic Parameter Levels

The levels of the sera TC, TG, LDL-C and HDL-C were tested using commercial enzymatic kits following the instructions on the kits [48].

### 3.10. Statistical Analysis

Data analyses were performed using Origin 2021b. The results were expressed as the mean ± standard deviation. The mean values among treatment groups were statistically analyzed by one-way analysis of variance (ANOVA) test. Turkey’s test was used to compare the results. *p* < 0.05 was considered statistically significant.

## 4. Conclusions

The exopolysaccharide PJ1-1 from the mangrove endophytic fungus *Penicillium janthinellum* N29 is a galactomannan comprising the →2)-α-d-Man*p*-(1→, →4)-α-d-Man*p*-(1→, →3)-β-d-Gal*f*-(1→ and →2)-β-d-Gal*f*-(1→ units. The branches consist of →3)-β-d-Gal*f*-(1→ units located at C-3 of the →2)-β-d-Gal*f*-(1→ unit. Compared to the MC group, PJ1-1 possessed a better ability to stimulate glucose metabolism, and improved the hypoglycemic efficiency of insulin and lipid metabolism in the T2DM mice. These data illustrated that PJ1-1 might be a potential anti-diabetic agent. Further investigation on the anti-diabetic mechanism of PJ1-1 is underway. Continuous studies will promote the development of anti-diabetic agents from marine microorganism.

## Figures and Tables

**Table 1:** Result of methylation analysis of PJ1-1.

Methylated Alditol Acetate	Molar Percent Ratio	Linkage Pattern
1,5-Di-*O*-acetyl-2,3,4,6-tetra-*O*-methyl mannitol	8.11	Man*p*-(1→
1,4-Di-*O*-acetyl-2,3,5,6-tri-*O*-methyl galactitol	7.50	Gal*f*-(1→
1,2,5-Tri-*O*-acetyl-3,4,6-tri-*O*-methyl mannitol	29.45	→2)-Man*p*-(1→
1,4,5-Tri-*O*-acetyl-2,3,6-tri-*O*-methyl mannitol	15.15	→4)-Man*p*-(1→
1,2,3,4-Tetra-O-acetyl-5,6-*O*-methyl galactitol	11.48	→2,3)-Gal*f* (1→
1,2,4-Tri-*O*-acetyl-3,5,6-di-*O*-methyl galactitol	13.01	→2)-Gal*f*-(1→
1,3,4-Tri-*O*-acetyl-2,5,6-di-*O*-methyl galactitol	15.30	→3)-Gal*f*-(1→

**Table 2:** ^1^H and ^13^C chemical shifts of PJ1-1.

Sugar Residues	Chemical Shifts (ppm) ^a^					
	H1/C1	H2/C2	H3/C3	H4/C4	H5/C5	H6/C6
→2)-α-D-Man*p*-(1→	5.29/102.19	4.15/79.67	4.01/71.64	3.71/71.39	3.66/68.35	3.76/62.48
→2,3)-β-D-Gal*f*-(1→	5.25/108.67	4.17/89.53	4.10/75.87	3.87/81.42	3.98/61.98	-/-
→2)-β-D-Gal*f*-(1→	5.23/106.25	4.17/89.53	4.03/71.64	3.87/83.23	3.78/61.98	-/-
β-D-Gal*f*-(1→	5.20/107.87	4.15/79.90	4.01/71.56	3.73/82.30	-/-	-/-
→4)-α-D-Man*p*-(1→	5.16/99.76	4.10/71.56	3.87/71.97	3.82/74.78	3.72/68.47	3.65/64.28
α-D-Man*p*-(1→	5.12/103.79	3.92/71.56	4.01/71.64	3.75/67.96	3.72/64.30	3.92/62.58
→3)-β-D-Gal*f*-(1→	5.08/109.31	4.18/82.61	4.07/79.78	3.77/83.67	3.72/64.30	-/-

^a^ Spectra were performed on an Agilent DD2 500 MHz NMR spectrometer. Chemical shifts are referenced to internal acetone at 2.225 ppm for ^1^H and 31.07 ppm for ^13^C. Man*p*: mannopyranose, Gal*f*: galactofuranose.

**Table 3:** Effect of PJ1-1 on fasting blood glucose level in the type 2 diabetes mellitus mice.

	Fasting Blood Glucose Level (mmol/L) ^a^					
	NC	MC	PC	PJ1-1-H	PJ1-1-M	PJ1-1-L
0 week	5.25 ± 0.18	18.21 ± 0.77 ^##^	20.15 ± 2.15 ^##^	20.67 ± 2.08 ^##^	21.02 ± 2.66 ^##^	22.14 ± 1.92 ^##^
1 week	5.19 ± 0.18	21.47 ± 1.10 ^##^	21.18 ± 2.15	21.28 ± 2.11	21.06 ± 2.21	20.89 ± 1.51
2 week	5.27 ± 0.32	21.50 ± 1.10 ^##^	21.12 ± 1.60	18.62 ± 1.04 **	19.59 ± 1.73	20.12 ± 1.54
3 week	5.19 ± 0.18	21.77 ± 0.89 ^##^	18.18 ± 0.99 **	16.60 ± 1.62 **	16.33 ± 1.75 **	18.82 ± 1.37 **
4 week	5.10 ± 0.25	22.13 ± 0.76 ^##^	14.40 ± 0.90 **	15.32 ± 1.44 **	16.77 ± 1.47 **	17.62 ± 0.97 **
5 week	5.19 ± 0.32	21.75 ± 1.36 ^##^	9.75 ± 1.34 **	12.58 ± 0.77 **	15.42 ± 1.58 **	16.91 ± 1.71 **

^a^ Values are mean ± SD (*n* = 6). ^##^ *p* < 0.01 vs. the normal control group; ** *p* < 0.01 vs. the model control group.

## Data Availability

Data presented in this study are available on request.
